# Alterations in DNA methylation/demethylation intermediates predict clinical outcome in chronic lymphocytic leukemia

**DOI:** 10.18632/oncotarget.20081

**Published:** 2017-08-09

**Authors:** Cristina Bagacean, Adrian Tempescul, Christelle Le Dantec, Anne Bordron, Audrey Mohr, Hussam Saad, Valerie Olivier, Mihnea Zdrenghea, Victor Cristea, Pierre-François Cartron, Nathalie Douet-Guilbert, Christian Berthou, Yves Renaudineau

**Affiliations:** ^1^ U1227 B Lymphocytes and Autoimmunity, University of Brest, INSERM, IBSAM, Labex IGO, Networks IC-CGO and REpiCGO from Cancéropôle Grand Ouest, Brest, France; ^2^ Laboratory of Immunology and Immunotherapy, Brest University Medical School Hospital, Brest, France; ^3^ Iuliu Hatieganu University of Medicine and Pharmacy, Cluj-Napoca, Romania; ^4^ Department of Hematology, Brest University Medical School Hospital, Brest, France; ^5^ Department of Hematology, ‘Ion Chiricuta’ Oncology Institute, Cluj-Napoca, Romania; ^6^ Inserm, U892, Epigenetics Network from Cancéropôle Grand Ouest, Nantes, France; ^7^ Laboratory of Cytogenetics, Brest University Medical School Hospital, Brest, France

**Keywords:** chronic lymphocytic leukemia, methylation, hydroxymethylation, TET, SAT1

## Abstract

Cytosine derivative dysregulations represent important epigenetic modifications whose impact on the clinical outcome in chronic lymphocytic leukemia (CLL) is incompletely understood. Hence, global levels of 5-methylcytosine (5-mCyt), 5-hydroxymethylcytosine (5-hmCyt), 5-carboxylcytosine (5-CaCyt) and 5-hydroxymethyluracil were tested in purified B cells from CLL patients (*n* = 55) and controls (*n* = 17). The DNA methylation ‘writers’ (DNA methyltransferases [*DNMT1/3A/3B*]), ‘readers’ (methyl-CpG-binding domain [*MBD2/4*]), ‘editors’ (ten-eleven translocation [*TET1/2/3*]) and ‘modulators’ (*SAT1*) were also evaluated. Accordingly, patients were stratified into three subgroups. First, a subgroup with a global deficit in cytosine derivatives characterized by hyperlymphocytosis, reduced median progression free survival (PFS = 52 months) and shorter treatment free survival (TFS = 112 months) was identified. In this subgroup, major epigenetic modifications were highlighted including a reduction of 5-mCyt, 5-hmCyt, 5-CaCyt associated with *DNMT3A*, *MBD2/4* and *TET1/2* downregulation. Second, the cytosine derivative analysis revealed a subgroup with a partial deficit (PFS = 84, TFS = 120 months), mainly affecting DNA demethylation (5-hmCyt reduction, *SAT1* induction). Third, a subgroup epigenetically similar to controls was identified (PFS and TFS > 120 months). The prognostic impact of stratifying CLL patients within three epigenetic subgroups was confirmed in a validation cohort. In conclusion, our results suggest that dysregulations of cytosine derivative regulators represent major events acquired during CLL progression and are independent from *IGHV* mutational status.

## INTRODUCTION

The clinical outcome of chronic lymphocytic leukemia (CLL) is heterogeneous with some patients remaining asymptomatic for decades, while others progress rapidly and need therapy soon after diagnosis [24]. Due to the development of novel targeted therapies in CLL, the overall survival of the patients has increased [3].In contrast, the criteria for treatment initiation have not evolved in the last decades alluding to the fact that determining the delay from diagnosis to treatment initiation represents the gold standard for evaluating disease progression [11]. Therefore, when using the treatment free survival (TFS) as end point, a long list of prognostic factors has been established such as the mutational status of the immunoglobulin heavy chain variable region (*IGHV*), cytogenetic alterations, CD38, and more recently, epigenetic modifications, which have been proposed to be the most accurate factor for disease progression [32, 33, 42]. Moreover, the notion that epigenetic factors are relevant for CLL is further supported by the observation that CLL tumor cells carry less genetic mutations than other adult cancers [30].

Epigenome-wide association studies (EWAS) have contributed to better understanding the significance of DNA methylation in CLL by showing: (1) global DNA hypomethylation combined with local hypermethylation in CLL B cells as observed in other cancers [22]; (2) a link between the methylome profile heterogeneity, an increased genetic complexity and adverse clinical outcome, thus suggesting some epigenetic modifications as an unfavorable and heritable trait [29, 35]; (3) a distinct DNA methylation signature between the two molecular *IGHV* subtypes in relation to naïve B cells and unmutated (UM)-CLL, or memory B cells and mutated (M)-CLL [27]; (4) that epigenetic patterns are similar between peripheral blood and lymph node compartments [4], while in contrast, the transcriptome is influenced by the B-cell receptor (BCR) activation in the microenvironment of the lymph node [13]; and finally (5) aberrant DNA methylation and specific chromatin accessible region overlap with gene loci encoding for risk factors [44]. In the CLL prone-mice model Eµ-*TCL1*, development of the disease is preceded by the appearance of important epigenetic modifications [7]. It has also been shown that mice with biallelic loss of the DNA methyltransferase *(DNMT)3A* develop a lymphoproliferative disease similar to CLL [17].

In general, DNA methylation is considered to be a tightly regulated and stable epigenetic mark. Therefore, modifications in DNA methylation have a major impact in cancer and certainly, CLL. Multiple pathways control DNA methylation. Biochemically, DNA methylation refers to the addition of a methyl subgroup to the fifth carbon of a cytosine by DNMT1/3A/3B resulting in generation of 5-methylcytosine (5-mCyt), and then, methyl-CpG binding domain (MBD) proteins are recruited to regulate chromatin behavior and gene expression. *DNMTs* and *MBD*s expression patterns were studied in CLL to a certain extent, mainly revealing that the mechanisms are complex and that other regulators are involved [26]. An active DNA demethylation process was recently described involving the Ten-Eleven Translocation (TET) enzyme family (TET1/2/3), which oxidizes 5-mCyt into 5-hydroxymethylcytosine (5-hmCyt) and subsequently, in a less efficient fashion, into 5-formylcytosine (5-fCyt) and 5-carboxylcytosine (5-CaCyt) with the use of α-ketoglutarate (α-KG), molecular oxygen and iron as cofactors [47]. *TET* expression varies in CLL patients but paradoxically it is lower expression that has been reported for patients with a reduced TFS [50], while others have reported an overexpression in CLL B cells compared to controls [19]. DNA methylation and active DNA demethylation processes are not restricted to DNMTs and TETs, respectively, as other models have demonstrated that 5-hmC can be enzymatically deaminated by activation-induced deaminase (AID)/apolipoprotein B editing complex (APOBEC) to 5-hydroxymethyluracil (5-hmU) which can then be processed by various DNA glycosylases, including MBD4, generating an unmodified cytosine [21, 31, 37]. Other events can modify methylation levels, such as an increase in spermidine/spermine N1-acetyltransferase (SAT1) which can promote an excessive consumption of S-adenosyl methionine (SAM), the methyl donor molecule leading to global DNA hypomethylation [5, 23, 34].

In the present study, we measured cytosine derivatives and associated them with the clinical outcome to define three subgroups with distinct disease courses. We also investigated DNA methylation regulators according to the 5-Cyt derivative profile in order to better understand key factors regulating DNA methylation and active DNA demethylation in CLL during disease progression.

## RESULTS

### CLL population

The study cohort consists of 55 untreated patients diagnosed with CLL according to the WHO classification criteria [46]. As listed in Table 1, the median age at diagnosis was 65 years (range 43–84) with a majority of CLL patients with Binet stage A disease 34/55 (60.7%),followed by Binet stage B 19/55 (33.9%) and C 3/55 (5.5%). The median progression free survival (PFS) and TFS of the studied cohort were 104 months and 120 months, respectively. The major prognostic markers associated with disease progression were analyzed including lymphocytosis, lymphocyte doubling time (LDT), *IGHV* mutational status, CD38 and cytogenetic factors. Patients were categorised into cytogenetic risk groups according to the following classification: low risk group - isolated del(13q); intermediate risk group - trisomy 12 or normal karyotype and fluorescence *in situ* hybridization (FISH) and high risk group - del(11q), del(17p) or complex karyotype [41].

**Table 1:** Characteristics of the patients

Characteristics	Value
Sex (male/female) - number of patients	37/19
Age at diagnosis - median [range]	65 [43–84]
Age at study entry - median [range]	72 [55–88]
Binet stage - number of patients (%)	
A	34/55 (60.7%)
B	19/55 (33.9%)
C	3/55 (5.4%)
Lymphocytosis (Giga/L) - median (± SEM)	51.6 (± 6.53)
Lymphocyte doubling time - median (months)*	24
*IGHV* mutational status - number of patients (%)	
Unmutated (≥ 98% homology)	10/48 (20.8%)
Mutated (< 98% homology)	38/48 (79.2%)
CD38 > 30% - number of patients (%)	14/55 (25%)
Cytogenetic risk category - number of patients (%)	
Low risk - Isolated del(13q)	17/55 (30.4%)
Intermediate risk- Trisomy 12, normal karyotype and FISH	15/55 (26.8%)
High risk - del(11q), del(17p), complex karyotype	12/55 (21.4%)
Progression free survival - median (months)*	104
Treatment free survival - median (months)*	120

Abbreviations: *IGHV*, immunoglobulin heavy chain variable region; FISH, fluorescence *in situ* hybridization; *, Kaplan-Meyer survival analysis.

### Cytosine (5-Cyt) derivatives and clinical outcome in CLL

**Figure 1:**
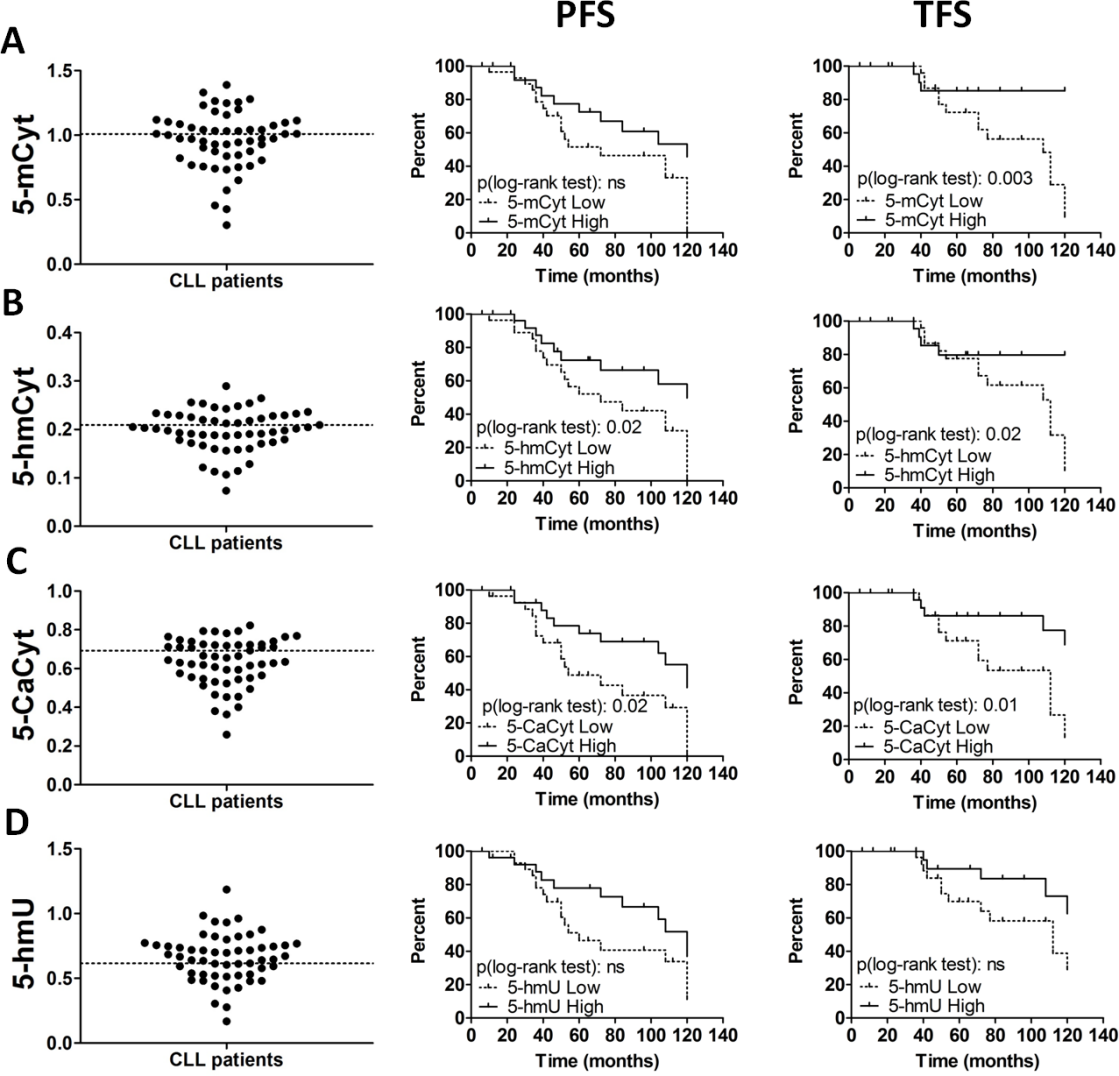
Global levels of cytosine derivatives in chronic lymphocytic leukemia (CLL) patients (**A**) 5-methylcytosine (5-mCyt), (**B**) 5-hydroxymethylcytosine (5-hmCyt), (**C**) 5-carboxylcytosine (5-CaCyt), and (**D**) 5-hydroxymethyluracil (5-hmU) were determined in purified B cells from 55 untreated CLL patients. ELISA results plots (left) for each 5-Cyt derivative and Kaplan-Meier plots depicting the progression free survival (PFS, middle) and treatment free survival (TFS, right) probability over time from diagnosis. ELISA results are expressed as indexes using a DNA reference sample for normalization (see Materials and methods section). The optimal cut-off, represented by dashed lines on the ELISA plots (left), was calculated using the profile likelihood method in a Cox regression model of TFS. Statistical significance was determined by log-rank test and *P* < 0.05 are indicated; ns, not significant.

As the DNA methylation and active DNA demethylation processes are dynamic and suspected to vary during disease evolution in CLL [35], four adapted enzyme linked immunosorbent assay (ELISA) techniques were performed in order to study the relationship between global DNA methylation (5-mCyt), on the one hand, and the active demethylation pathway (5-hmCyt, 5-CaCyt and 5-hmU), on the other hand. The ELISAs were performed on genomic DNA extracted from purified B cells of 55 CLL patients (Figure 1 - left part, Table 1) and patients were dichotomized into high *versus* low levels using the profile likelihood method in a Cox regression model of TFS for optimal cut-off identification.

Regarding PFS (Figure 1 - middle part), the Kaplan-Meier log-rank analysis revealed significant differences for the two active DNA demethylation marks 5-hmCyt (*P =* 0.02) and 5-CaCyt (*P =* 0.02). When TFS was taken into consideration (Figure 1 - right part), the impact of the DNA methylation marks, 5-mCyt, was more important (median TFS was 108 months in low 5-mCyt CLL group *versus* > 120 months in high 5-mCyt CLL group, *P =* 0.003), followed by the active DNA demethylation marks, 5-hmCyt (112 months *versus* > 120 months, *P =* 0.02) and 5-CaCyt (112 months *versus* > 120 months, *P =* 0.01). For 5-hmU, differences did not reach significance regarding PFS and TFS. Accordingly, the removal of the hydroxymethyl group *via* 5-hmU seems not to be a highly implicated pathway in the dysregulated dynamics of active DNA demethylation in CLL. Therefore, the latter parameter was no longer considered for further analysis.

### Interconnection between DNA methylation and active DNA demethylation processes in CLL B cells

**Figure 2:**
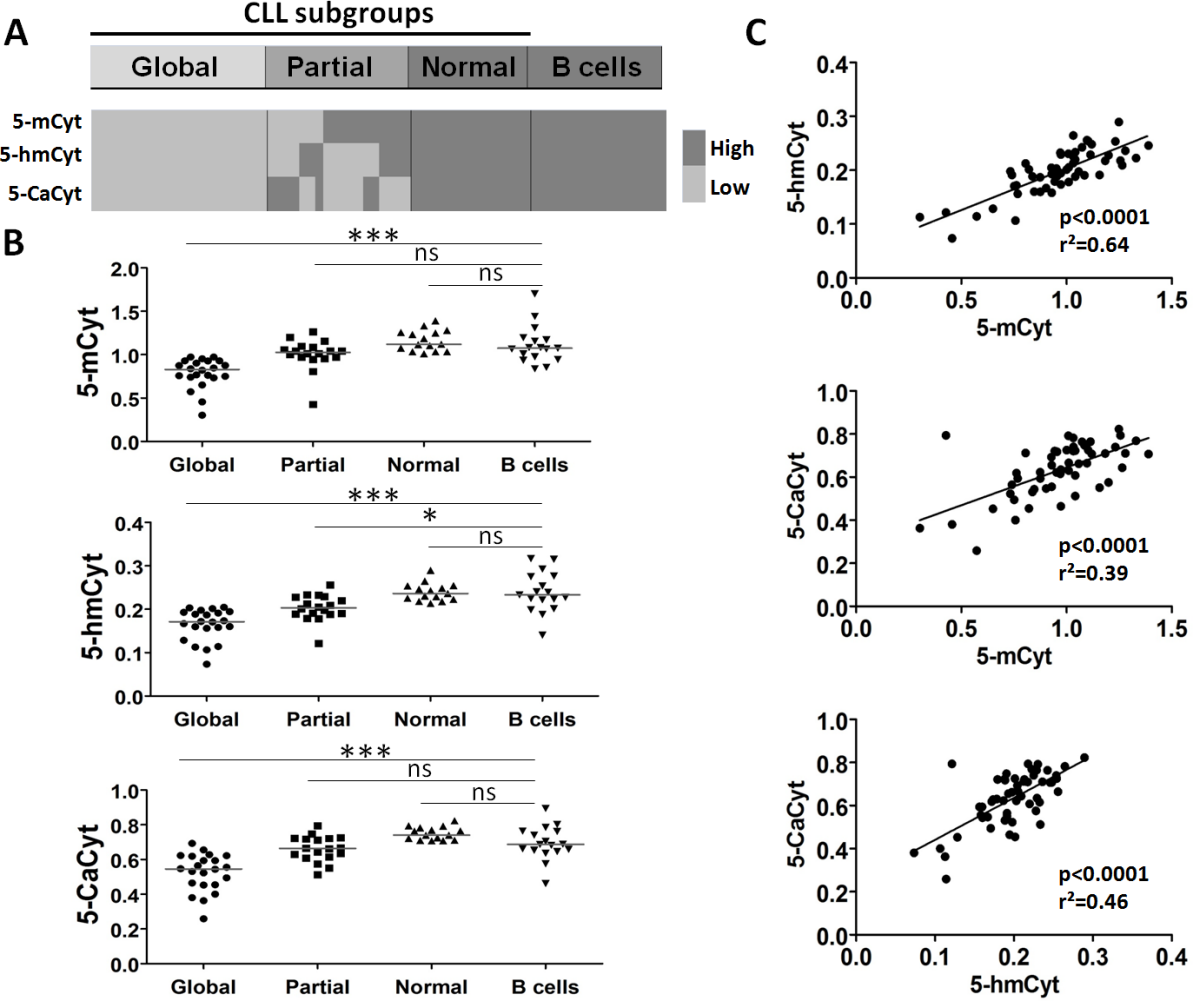
Analysis of cytosine (5-Cyt) derivatives in chronic lymphocytic leukemia (CLL) B cells defines distinct subgroups (**A**) The graphical representation indicates the presence of three subgroups by comparing CLL B cells and control B cells with high and low levels of global 5-methylcytosine (5-mCyt), 5-hydroxymethylcytosine (5-hmCyt), and 5-carboxylcytosine (5-CaCyt) as determined in Figure 1. The 3 subgroups: global deficiency in all 5-Cyt derivatives (global, *n* = 22), partial deficiency in 1 or 2 of the 5-Cyt derivatives (partial, *n* = 18), and 5-Cyt derivative normal content (normal, n=15) similar to healthy control B cells (B cells, *n* = 17). (**B**) 5-Cyt derivative levels according to the 3 CLL subgroups and control B cells. The median value is indicated (line), and statistical differences of the comparison with control B cells are indicated as follows: (***) *P* < 0.001; (**) 0.001 < *P* < 0.01; (*) 0.01< *P* < 0.05; ns, not significant. (**C**) Linear regression analysis between 5-mCyt, 5-hmCyt and 5-CaCyt. The *P* and r^2^ values are indicated.

Based on the observation that an association was observed between PFS and the dysregulation of the active DNA demethylation process (5-hmCyt and/or 5-CaCyt low), on the one hand, and between TFS and a reduction in the three 5-Cyt derivatives, on the other hand, we hypothesized that both mechanisms are interconnected in CLL B cells. Therefore, we further segregated CLL patients according to the combined analysis of 5-mCyt, 5-hmCyt and 5-CaCyt. Three subgroups were revealed as follows: CLL patients with a global deficiency in 5-Cyt derivatives (Global, *n =* 22), CLL patients with 1 or 2 deficient 5-Cyt derivatives (Partial, *n =* 18) and CLL patients with a 5-Cyt derivative content similar to normal B cells (Normal, *n =* 15) (Figure 2A). Comparison with control B cells (B cells) from 17 subjects (Figure 2B), revealed for the subgroup with a global deficit the diminishing of 5-mCyt (index: 0.83 ± 0.17 in the CLL Global subgroup *versus* 1.07 ± 0.05 in control B cells, *P* < 10^-4^), 5-hmCyt (index: 0.17 ± 0.04 in the CLL Global subgroup *versus*0.23 ± 0.01 in control B cells, *P* < 10^–4^) and 5-CaCyt (index: 0.54 ± 0.10 in the CLL Global subgroup *versus* 0.69 ± 0.02 in control B cells, *P* < 10^–4^). In the subgroup with a partial deficit, decrease was restricted to 5-hmCyt (index: 0.20 ± 0.03 in the CLL Partial subgroup *versus* 0.23 ± 0.01 in control B cells, *P =* 0.01), while no differences were reported for CLL patients from the Normal subgroup compared to control B cells. Of note, 5-mCyt, 5-hmCyt and 5-CaCyt levels were highly correlated (*P* < 0.0001 for all; Figure 2C), which highlights the interconnections between the different processes.

### Epigenetic regulator expression is independent from *IGHV* mutational status and the levels are similar to memory B cells

**Figure 3:**
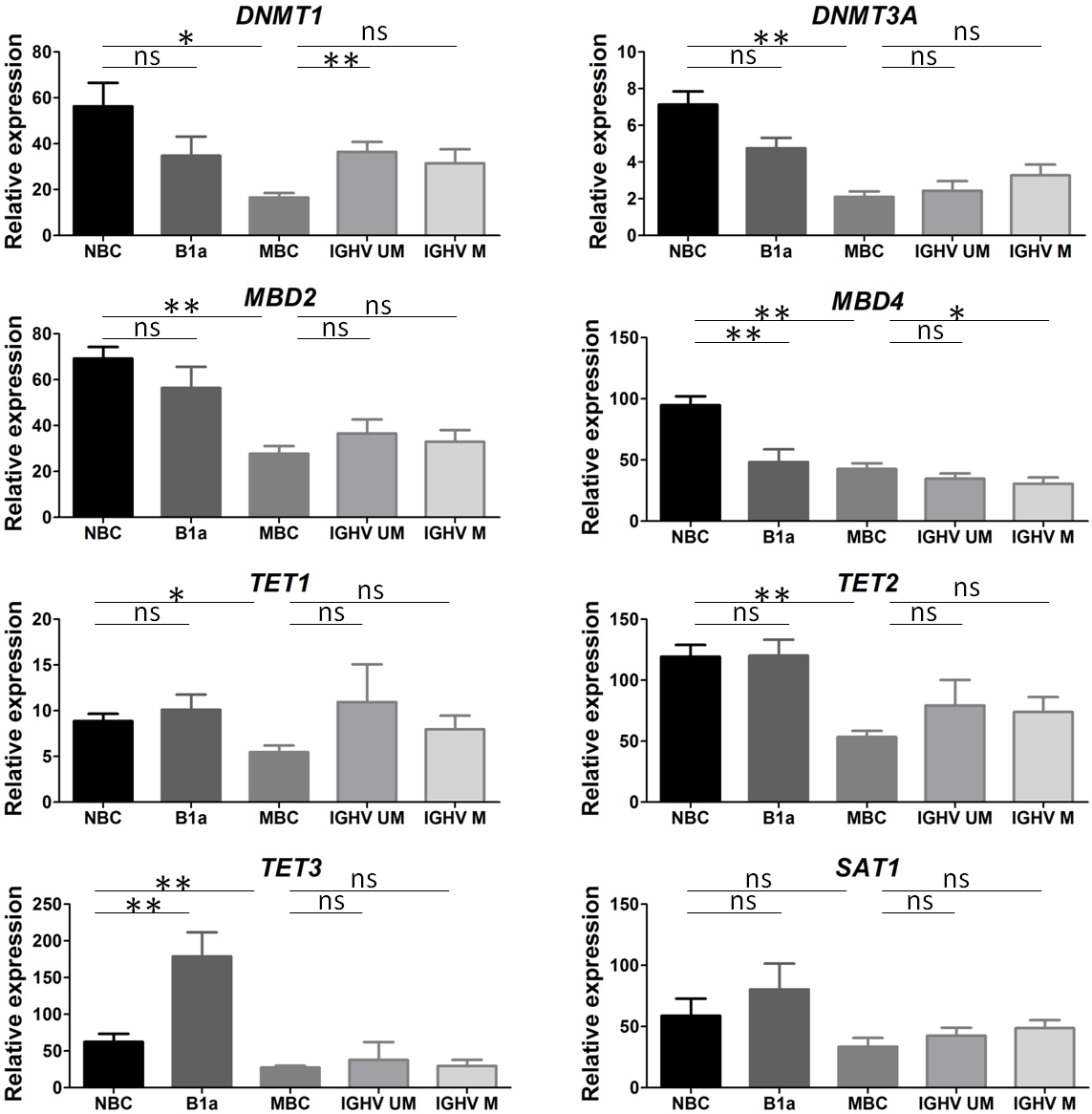
Epigenetic regulator expression varies according to B cell development Naïve B cells (NBC), CD5+ B1 cells (B1a) and memory B cells (MBC) were purified from peripheral blood of 6 healthy controls. Chronic lymphocytic leukemia (CLL) patients tested for immunoglobulin heavy chain variable region (*IGHV*) mutational status were divided into unmutated *IGHV* gene (UM > 98% homology from germline sequences) and mutated *IGHV* gene (M < 98% homology). Epigenetic regulators (*DNMT1/3A, MBD2/4, TET1/2/3, SAT1*) were tested by real time PCR for 6 *IGHV* UM and 20 *IGHV* M CLL patients and expressed relative to *GAPDH*. Values are represented as means ± SEM. The level of significance indicated by asterisks represents the comparison with NBC or MBC, and statistical differences are indicated as follows: (**) 0.001 < *P* < 0.01; (*) 0.01 < *P* < 0.05; ns, not significant.

In order to explain differences between the three 5-Cyt derivative subgroups, we first suspected an innate process due to important variations during germinal B cell differentiation [28]. For getting insight into the mechanisms controlling DNA methylation and active DNA demethylation in CLL, DNA methylation ‘writers’, *DNMT1/3A/3B*; ‘readers’, *MBD2/4*; ‘editors’, *TET1/2/3*; and the ‘modulator’ *SAT1,* were tested by real time quantitative (RTq)-polymerase chain reaction (PCR) on purified peripheral blood naïve B cells, CD5 B1 cells and memory B cells from 6 healthy controls and B cells from 26 CLL patients for whom we have also determined the *IGHV* mutational status (Figure 3). *DNMT3B*, which was expressed at low levels in the CLL and control B cell subsets, was excluded from further analysis.

Except for *SAT1* which was stable, the transition from naïve B cells to memory B cells was associated with a downregulation of epigenetic regulators (*P* < 0.05 for all). When naïve B cells and CD5+ B1a cells were taken into consideration for analysis, the main differences were related to *MBD4* downregulation (*P =* 0.02) and *TET3* upregulation (*P =* 0.004) in CD5+ B1a cells.

Next, CLL B cells were subdivided into two groups according to their *IGHV* mutational status as they originate either from B cells which have not undergone differentiation in the germinal centers (*IGHV* UM), or from post-germinal center B cells (*IGHV* M). In contrast to control B cells, for tumor B cells the hypothesis of epigenetic modifications related to the *IGHV* mutational status has to be rejected based on several observations. First, the distribution of *IGHV* UM and M CLL patients was similar between the three 5-Cyt derivative subgroups (5/14 in the Global subgroup, 2/14 in the Partial subgroup, and 3/10 in the Normal subgroup) (Table 2),and no difference was observed with regards to the 5-Cyt derivatives according to the *IGHV* mutational status (Supplementary Figure 1). Second (Figure 3), the transcript levels for the 8 epigenetic regulators were similar between *IGHV* UM and M CLL B cells. Moreover and third, in both *IGHV* UM and M CLL B cells, the epigenetic regulator profiles were close to the memory B cell profile. Indeed, when comparing CLL B cells to memory B cells, differences were related to the *IGHV* UM CLL group for *DNMT1* (*P =* 0.0025) and *TET2* (*P =* 0.04); and to the *IGHV* M CLL group for *MBD4* (*P =* 0.03).

**Table 2:** The relevance of clinicobiological prognostic factors according to the three cytosine derivative subgroups

Prognostic factors and clinical outcome	Global	Partial	Normal	Statistics (p)
Age at diagnosis - median [range]	67.5 [53–78]	65.5 [54–84]	56 [43–79]	ns
Age at study entry - median [range]	72.5 [59–87]	72.5 [61–88]	68 [55–83]	ns
Binet stage - number of patients (%)				ns
A	10/22 (45.5%)	12/18 (66.7%)	12/15 (80%)	
B and C	12/22 (54.5%)	6/18 (33.3%)	3/15 (20%)	
Lymphocytosis (Giga/L) - median (± SEM)	81.6 (± 12.06)	33 (± 6.31)	42.9 (± 8.68)	0.001
Lymphocyte doubling time from diagnosis (months) *	19	36	36	0.01
*IGHV* mutational status - number of patients (%)				ns
Unmutated (≥ 98% homology)	5/19 (10.42%)	2/16 (4.17%)	3/13 (6.25%)	
Mutated (< 98% homology)	14/19 (29.17%)	14/16 (29.17%)	10/13 (20.83%)	
Cytogenetics - number of patients (%)				0.005
Low risk - Isolated del(13q)	2/15 (9.5%)	10/17 (58.8%)	5/11 (38.5%)	
Intermediate risk - Trisomy 12, normal karyotype and FISH	7/15 (33.3%)	5/17 (29.4%)	2/11 (15.4%)	
High risk - del(11q), del(17p), complex karyotype	6/15 (28.6%)	2/17 (11.8%)	4/11 (30.8%)	
CD38 > 30% - number of patients (%)	7/22 (31.8%)	5/18 (27.8%)	2/15 (13.3%)	ns
5-mCyt Index - median (± SEM)	0.83 (± 0.04)	1.03 (± 0.04)	1.12 (± 0.03)	*p* < 0.0001
5-hmCyt Index - median (± SEM)	0.17 (± 0.008)	0.20 (± 0.007)	0.24 (± 0.005)	*p* < 0.0001
5-CaCyt Index - median (± SEM)	0.54 (± 0.02)	0.66 (± 0.02)	0.74 (± 0.01)	*p*< 0.0001
Progression free survival - median (months)*	52	84	Undefined	0.01
Treatment free survival - median (months)*	112	120	Undefined	0.01

Abbreviations: ns, not significant; *IGHV*, immunoglobulin heavy chain variable region; FISH, fluorescence *in situ* hybridization; 5 mCyt, 5-methylcytosine; 5-hmCyt, 5-hydroxymethylcytosine; 5-CaCyt, 5-carboxylcytosine; *, Kaplan-Meyer survival analysis. Statistical analysis was assessed using One-Way Anova test, Fisher’s exact test, or Log-rank analysis.

### Epigenetic regulator expression according to the cytosine derivative subgroups

**Figure 4:**
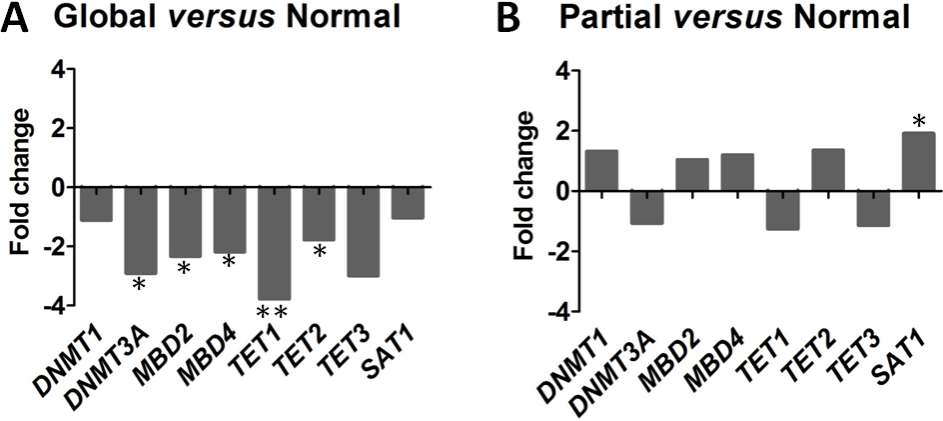
Distinct epigenetic regulator profiles according to the 3 cytosine derivative subgroups Epigenetic regulators are tested by real time PCR and expressed relative to *GAPDH*: the DNA methylation ‘writers’, *DNMT1/3A*; ‘readers’, *MBD2/4*; ‘editors’, *TET1/2/3*; and the ‘modulator’ *SAT1*. (**A**) Fold changes of epigenetic regulators between the chronic lymphocytic leukemia (CLL) global subgroup (*n* = 9) compared to the CLL normal subgroup (*n* = 13). (**B**) Fold changes of epigenetic regulators between the CLL Partial subgroup (*n* = 11) compared to the CLL Normal subgroup. Statistical differences between Global and Partial subgroups *versus* Normal subgroup were assessed using the non-parametric Mann-Whitney test and *P* < 0.05 are indicated as follows: (***) *P* < 0.001; (**) 0.001 < *P* < 0.01; (*) 0.01 < *P* < 0.05.

As the epigenetic profiles are not related to the *IGHV* mutational status, we proceeded in the analysis of the mechanisms regulating DNA methylation and active DNA demethylation in the Global (*n =* 9) and Partial (*n =* 11) subgroups by comparing them to the Normal subgroup (*n =* 13) (Figure 4A, 4B).

When using the non-parametric Mann-Whitney *U* test to compare the subgroups, patients from the subgroup with a global deficiency were characterized by a reduction in the ‘writer’ *DNMT3A* (*P =* 0.01), the ‘readers’ *MBD2* (*P =* 0.03), *MBD4* (*P =* 0.03) and the ‘editors’ *TET1* (*P =* 0.007) and *TET2* (*P =* 0.01). In contrast, the subgroup with a partial deficiency differed from the Normal subgroup through only one epigenetic regulator, *SAT1*, which was overexpressed (*P =* 0.02; fold change × 1.9).We conclude from this experiment that a defect of both DNA methylation and active DNA demethylation (Global subgroup) is associated with a downregulation of most of the key epigenetic regulators, while for the Partial subgroup the process is more complex as only the overexpression of the DNA methylation ‘modulator’ SAT1 was observed.

### Epigenetic regulator expression during disease progression

**Figure 5:**
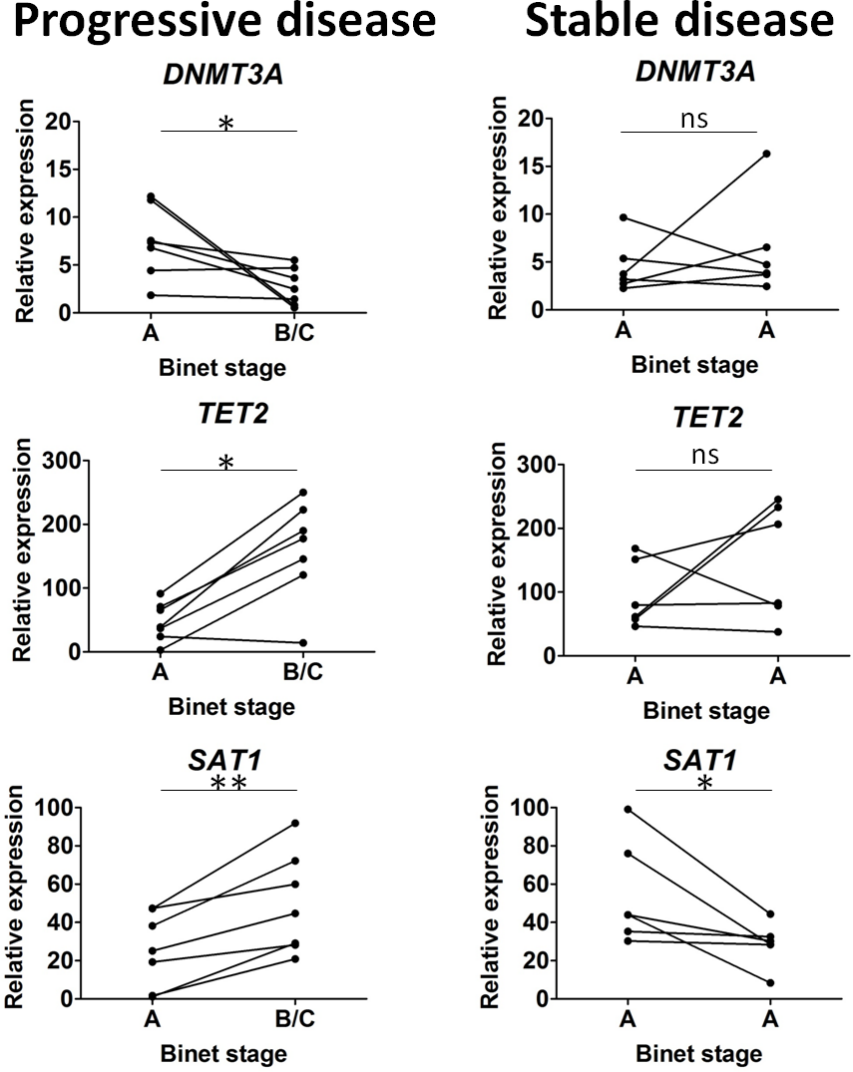
Epigenetic regulator variations during disease progression Sequential samples were selected either from chronic lymphocytic leukemia (CLL) patients evolving from Binet stage A to Binet stage B/C (progressive disease, *n* = 7), or from CLL patients in Binet stage A at the 2 time points (stable disease, *n* = 6). Among the 8 epigenetic regulators tested, only *DNMT3A, TET2* and *SAT1* presented significant differences and are represented in the figure. Statistical differences are indicated by asterisks: (**) 0.001 < *P* < 0.01; (*) 0.01 < *P* < 0.05, ns, not significant.

Next, we further tested epigenetic regulator mRNA expression levels at two time points in patients with progressive disease (stage Binet A and stage Binet B/C, *n =* 7) and patients with stable disease (Binet stage A at the two time points, *n =* 6). The second time point corresponds to patients included in the training cohort and the mean interval between the two time points was 18 months (range 12-36 months). Among the 8 epigenetic regulators tested (Figure 5), a statistically significant modification between the two time points was observed in progressive disease patients for *DNMT3A* (downregulation, *P =* 0.03), *TET2* (upregulation, *P =* 0.03) and *SAT1* (upregulation, *P =* 0.01). In the stable disease group, differences concerned *SAT1*, which was, contrarily to the progressive disease patients, downregulated (*P =* 0.03). Altogether, these results indicated that transcriptome variations reported during CLL evolution include at least three key epigenetic regulators: *DNMT3A*, *TET2* and *SAT1* [56].

### CLL patient subgroups and clinical outcome

We next analyzed the relationship between established prognostic markers and the three 5-Cyt derivative subgroups identified (Table 2).

Regarding the CLL subgroup with a global deficiency, a more aggressive profile was highlighted, as patients were characterized by an elevated lymphocytosis (81.6 ± 56.6 Giga/L in the Global subgroup *versus* 33.0 ± 26.8 Giga/L and 42.9 ± 33.7 Giga/L in the Partial and Normal subgroups, respectively; *P =* 0.001) and a reduced LDT (19 ± 10.5 months in the Global subgroup *versus* 36 ± 25 months in the Partial subgroup and 36 ± 23 months in the Normal subgroup; *P =* 0.01). Additionally, the cytogenetic abnormalities which define the intermediate and high risk CLL patients were better represented in the subgroups with a global and partial 5-Cyt derivative deficit (*P =* 0.005).

Next, the prognostic power of the 3 defined 5-Cyt derivative subgroups of CLL patients was evaluated first on PFS (Supplementary Figure 2). As depicted in the Kaplan-Meier curves (Supplementary Figure 2A), CLL patients from the Global subgroup had the shortest median PFS of 52 months (Global *versus* Normal, *P =* 0.003). The Partial subgroup is intermediate with a median PFS of 84 months, while for the Normal subgroup, the median survival intervals were not reached (> 120 months). In univariate analysis, the following variables were associated with a shorter PFS: elevated *SAT1* transcripts (*P =* 0.04), advanced age at diagnosis (*P =* 0.009), higher lymphocytosis (*P =* 0.008), shorter LDT (*P =* 0.003) and CD38 levels ≥ 30% (*P =* 0.0007).

**Figure 6:**
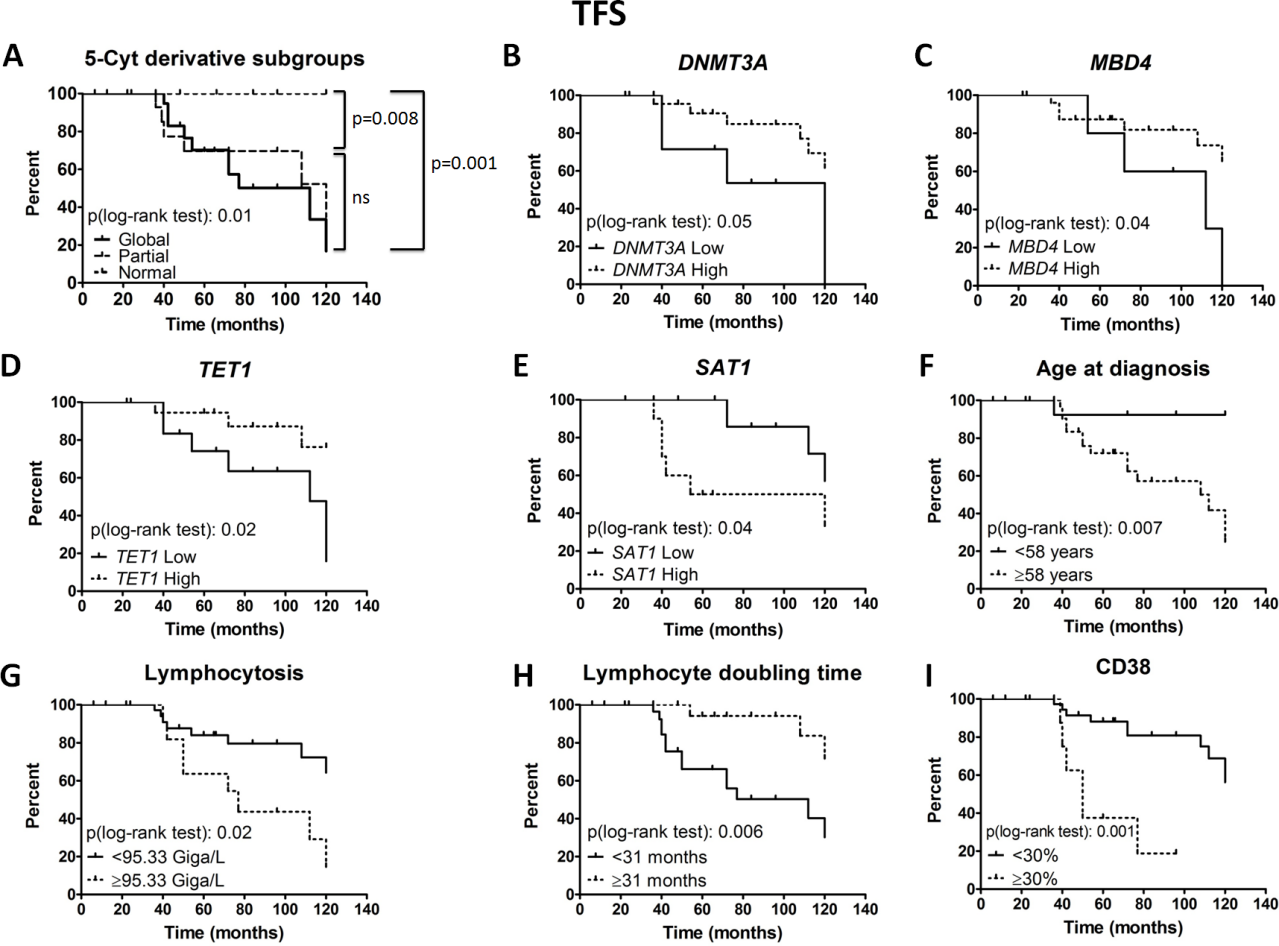
Prognostic power of epigenetic subgroups and regulators Treatment free survival (TFS) Kaplan-Meier curves are depicted for the 3 cytosine derivative subgroups (**A**), *DNMT3A* (**B**), *MBD4* (**C**), *TET1* (**D**), *SAT1* (**E**), age at diagnosis (**F**), lymphocytosis (**G**), lymphocyte doubling time from diagnosis (**H**) and CD38 (**I**). Epigenetic regulator expression patterns were measured by real time PCR and normalized to *GAPDH*. The Cox regression model of TFS was used to identify the optimal cut-off level in order to dichotomize CLL patients into high *versus* low levels, except for CD38. Statistical differences between the curves were calculated using the log-rank test.

Finally, regarding TFS (Figure 6), the median times to initial therapy were 112 months, 120 months and > 120 months for the CLL Global, Partial and Normal subgroups, respectively (Global *versus* Normal, *P =* 0.001). In univariate analysis, significant associations with shorter TFS were related to a reduction of *DNMT3A* (*P =* 0.05), *MBD4* (*P =* 0.04), *TET1* (*P =* 0.02) and LDT (*P =* 0.006); and to an increase of *SAT1* (*P =* 0.04), age at diagnosis (*P =* 0.007), lymphocytosis (*P =* 0.02) and CD38 (*P =* 0.001); We also performed a multivariate analysis, and the 5-Cyt derivatives emerged as independent prognostic parameters of TFS.

### Validation with an independent cohort

**Figure 7:**
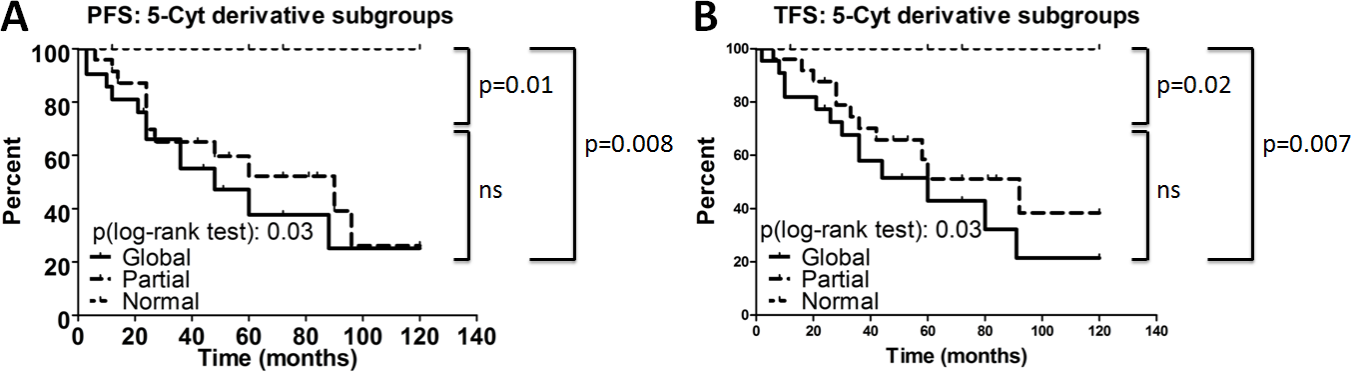
Prognostic power of epigenetic subgroups for the validation cohort Progression free survival (PFS) **(A)** and treatment free survival (TFS) **(B)** Kaplan-Meier curves are depicted for the 3 cytosine derivative subgroups (Global: *n* = 22; Partial: *n* = 26; Normal: *n* = 8) in the validation cohort. Statistical differences between the curves were calculated using the log-rank test.

The ability of stratifying CLL patients in epigenetic subgroups according to the 5-Cyt derivative content was confirmed in a separate group of 56 CLL patients (Table 3). By using the cut-offs previously determined with the Cox regression model for TFS, we classified these cases within a subgroup with a global deficit (*n =* 22), a subgroup with a partial deficit (*n =* 26) and a subgroup similar to control B cells (*n =* 8). As depicted in the Kaplan-Meyer curves from Figure 7A, the subgroup with a global deficit had the shortest median PFS (48 months), followed by the Partial deficit subgroup (90 months), while in the Normal subgroup, the median survival intervals were not reached (> 120 months) (Global *versus* Normal, *P =* 0.008). Concerning TFS (Figure 7B), the Global deficit subgroup had the shortest time to treatment initiation (60 months), followed by the Partial deficit subgroup (92 months) and the Normal subgroup (> 120 months) (Global *versus* Normal, *P =* 0.007). Herein, the PFS and TFS results on the validation cohort are similar to those obtained in the training series.

**Table 3:** The relevance of clinicobiological prognostic factors according to the three cytosine derivative subgroups in the validation cohort

Prognostic factors and clinical outcome	Global	Partial	Normal	Statistics (p)
Age at diagnosis - median [range]	62.5 [43–77]	62.5 [33–83]	67.4 [60–77]	ns
Age at study entry - median [range]	67.4 [45–85]	66.9 [33–87]	74.5 [61.89]	ns
Binet stage - number of patients (%)				ns
A	11/22 (50%)	15/26 (57.7%)	8/8 (100%)	
B and C	11/22 (50%)	11/26 (42.3%)	0/8 (0%)	
Lymphocytosis (Giga/L) - median (±SEM)	37.36 (± 11.96)	28.01 (± 5.01)	17.82 (± 3.55)	0.03
Lymphocyte doubling time from diagnosis (months)*	12	14.5	48	ns
*IGHV* mutational status - number of patients (%)				ns
Unmutated (≥ 98% homology)	5 (10.42%)	2 (4.17%)	3 (6.25%)	
Mutated (< 98% homology)	14 (29.17%)	14 (29.17%)	10 (20.83%)	
Cytogenetic risk category - number of patients (%)				0.005
Low risk - Isolated del(13q)	7/17 (41.2%)	11/20 (55%)	5/8 (62.5%)	
Intermediate risk- Trisomy 12, normal karyotype and FISH	0/17 (0%)	5/20 (25%)	3/8 (37.5%)	
High risk - del(11q), del(17p), complex karyotype	10/17 (58.8%)	4/20 (20%)	0/8 (0%)	
CD38 > 30% - number of patients (%)	5/20 (25%)	7/25 (28%)	2/8 (25%)	ns
5-mCyt Index - median (± SEM)	0.88 (± 0.02)	0.98 (± 0.03)	1.07 (± 0.03)	*p* < 0.0001
5-hmCyt Index - median (± SEM)	0.18 (± 0.007)	0.21 (± 0.003)	0.21 (± 0.003)	*p* < 0.0001
5-CaCyt Index - median (± SEM)	0.60 (± 0.01)	0.65 (± 0.01)	0.63 (± 0.01)	ns
Progression free survival - median (months)*	48	90	Undefined	0.03
Treatment free survival - median (months)*	60	92	Undefined	0.03

Abbreviations: ns, not significant; *IGHV*, immunoglobulin heavy chain variable region; FISH, fluorescence *in situ* hybridization; 5mCyt, 5-methylcytosine; 5-hmCyt, 5-hydroxymethylcytosine; 5-CaCyt, 5-carboxylcytosine; *, Kaplan-Meyer survival analysis. Statistical analysis was assessed using One-Way Anova test, Fisher’s exact test, or Log-rank analysis.

The relationship between the 5-Cyt subgroups and CLL established prognostic markers was also analyzed for the validation cohort (Table 3). In the subgroups of patients with a global and a partial deficit, the proportion of Binet stage B and C disease is significantly higher compared to the Normal subgroup (*P =* 0.03). The more aggressive profile for these two subgroups was further confirmed by increased lymphocytosis (*P =* 0.03) and by the greater proportion of the CLL patients presenting a high cytogenetic risk (*P =* 0.005). Altogether, these data validate the ability of a 5-Cyt derivative content stratification to predict CLL outcome.

## DISCUSSION

In this study, 5-Cyt derivatives were characterized in purified CLL B cells from 55 untreated patients in the training cohort and 56 patients in the validation cohort, in order to better understand the dysregulated epigenetic mechanisms with repercussions on disease progression. Results generated from the present study indicated that: (1) 5-Cyt derivative levels in CLL B cells have a critical impact on disease progression and initial treatment delay in CLL; (2) DNA methylation and active demethylation data analysis classifies CLL patients into 3 subgroups: a worst prognosis subgroup of patients with a defect of both DNA methylation and active DNA demethylation (Global subgroup) associated with a downregulation of most of the key epigenetic regulators, the Partial subgroup, with an altered active DNA demethylation process associated with *SAT1* overexpression, and the Normal subgroup which is epigenetically similar to controls and has a stable disease course; (3) disease progression is associated with dynamic modifications in epigenetic regulator expression, particularly *DNMT3A, TET2* and *SAT1*.

In terms of prognosis, determining global 5-Cyt derivatives related to DNA methylation (5-mCyt) and active DNA demethylation (5-hmCyt and 5-CaCyt) represent an accurate tool for evaluating disease progression. However, it should be kept in mind that global 5-Cyt derivative studies using ELISA are only able to reveal important quantitative variations but not qualitative variations in contrast to EWAS studies that are able to demonstrate that epigenetic differences are more marked in the promoters in *IGHV* UM than what is observed in *IGHV* M CLL B cells [27, 39]. Aberrant promoter DNA methylation changes have been established in CLL, resulting in silencing of tumor suppressor genes (e.g. Wnt pathway regulators) and reactivation of genes involved in apoptosis, cell proliferation and prognostic markers (e.g. *NFATc1* and *LPL*). Testing global DNA methylation in the repetitive elements Alu, LINE-1 and satellite DNA sequences (Sat-α) in CLL, Hoxha *et al.* have observed a link between global DNA hypomethylation and disease progression, a lower TFS, a shorter telomere length and an increase in chromosome instability. Contrarily, no associations were reported with *IGHV* mutational status, CD38 and ZAP70 expression, which is consistent with our observations [12, 20]. Furthermore, and similar to our study, previous analyses have failed to show a correlation between *DNMT* levels and global DNA methylation levels, on one hand, and between *TET* and global DNA hydroxymethylation levels, on the other hand [12, 50]. The lack of association could be most likely due to complex mechanisms (see below).

Exploring transcript levels in CLL is challenging as no consensus has emerged to define the normal counterpart of CLL B cells. Peripheral blood total B cells, peripheral blood B cell subsets (naïve, memory), CD5+ B1a cells from umbilical cord blood, peripheral blood or tonsil and CD5 transfected B cell lines have been used in order to uncover CLL specific modifications [15, 16, 43].This key point may explain, in part, the conflicting results reported in CLL when exploring *DNMT* [26, 27] and *TET* expression [19, 50]. Based on the well described process of epigenetic specialization during B cell lineage development [36], CLL B cells were subdivided according to their *IGHV* mutational status and compared to naïve B cells, CD5+ B1a cells and memory B cells. Regarding the epigenetic regulator profiles, results from our study revealed important changes during normal B cell differentiation and a more homogeneous profile in CLL B cells. Overall, the CLL epigenetic regulator profile has also been shown to be more related to memory B cells and independent from the *IGHV* mutational status. This is consistent with the observation that gene expression profiles resemble more the typical features of memory B cells [25] and with the report that the *IGHV* M CLL B cell transcriptome progressively evolves to a sub-network similar to that of the *IGHV* UM CLL B cell transcriptome before therapy [8]. To further explore an acquired process, epigenetic regulators were tested at two time points during disease progression from Binet stage A to Binet stage B/C. Remarkably such analysis revealed that disease evolution affects pathways controlling both DNA methylation (*DNMT3A, SAT1*) and active DNA demethylation (*TET2*). However, upstream pathways controlling DNA methylation and active DNA demethylation have not yet been deciphered.

Regarding DNA methylation, the DNA methylation ‘writer’ *DNMT3A* is the gene significantly downregulated in the 5-Cyt Global subgroup, which is in agreement with the report that *DNMT3A* is in the top 1% of the genes downregulated in CLL [49]. It has been further documented in the *DNMT3A* knockout (KO) CLL-mice model that *DNMT3A* gene expression controls global DNA methylation patterns in haematopoietic stem cells, and in addition, *DNMT3A* was shown to be critical for B cell and CD8+ T cell development [18, 40, 45]. Therefore, it is not surprising that an epigenetic reprogramming is described in CD8+ T cells from CLL patients, which is associated with an inverted CD4/CD8 ratio and a poor outcome [53]. In CLL B cells from patients with aggressive disease, mutated *NOTCH1* and TCL1 can act as DNA methylation inhibitors by interacting with DNMT3A at the protein level [2, 38]. Such an interaction was not explored in our study as patients with the most aggressive profile (Global subgroup) were characterized by *DNMT3A* downregulation. We also showed that a downregulation of *DNMT3A* occurs during disease progression and that low levels of *DNMT3A* were associated with an early treatment initiation. In addition to *DNMT3A*, we further analyzed the expression of *SAT1*, which is part of a complex system, causing excessive consumption of SAM, the 5-Cyt methyl donor molecule [5, 34]. We found that *SAT1* was upregulated when the patients progress from Binet stage A to Binet stage B/C, and contrarily, a downregulation was observed in patients with stable disease. In addition, *SAT1* was the only factor increased when comparing the 5-Cyt Partial subgroup with the Normal subgroup. Thus, it is not unexpected that elevated levels of *SAT1* were associated with a lower PFS and TFS, suggesting therefore that monitoring *SAT1* could be helpful in order to predict disease outcome.

Concerning active DNA demethylation and patients from the Partial subgroup, we uncovered a previously unappreciated role of active DNA demethylation intermediates in CLL outcome. A dysregulated active DNA demethylation process in CLL is further supported by the report of Hernández-Sánchez et al. [19], and by our observation that *TET2* levels were increased in patients with progressive disease. However, according to Van Damme *et al.* [50], it was a reduction of *TET2* that was associated with reduced PFS in CLL. We failed to confirm such an assertion but report an association between lower expression of *TET1* and reduced PFS. We also observed in the most aggressive disease profile, the Global subgroup, that *TET2* was downregulated together with *DNMT3A*, *TET1* and *MBD2/4* leading to the triple deficit in 5-mCyt, 5-hmCyt and 5-CaCyt. One explanation is that distinct epigenetic profiles can lead to lymphoproliferative disease and disease progression in CLL, as supported by knock-in (KI) or KO experiments. *TET2* overexpression in KI mice was shown to induce B cell reprogramming [10, 14], while the loss of *TET2* induces myeloid malignancies such as chronic myelomonocytic leukemia in KO mice [6]. The *TET1/2* double-KO (DKO) mice develop B cell malignancies, and in two-thirds of the cases, the immunophenotype is close to CLL B cells [55]. Cells from *TET1/2* DKO mice present an increase in 5-mCyt and a reduction in 5-hmCyt content [52]. In humans, *TET1* and *TET2* have been previously shown to be concomitantly downregulated in B cell acute lymphoblastic leukemia and to have overlapping functions in B cell development and leukemogenesis [55]. The Global subgroup of CLL patients, who are characterized by *TET1/2* downregulation associated with *DNMT3A* downregulation, display decrease of all 5-Cyt derivatives. The impact of the dual gene loss *DNMT3A/TET2* has also been investigated recently, and it induces the development of multiple lymphoid diseases [54]. In CLL patients from the Global group, further experiments are required to better understand how the tumor repressors *DNMT3A*, *TET1/2* but also *MBD2/4* are dysregulated, and the consequences of this repression on disease progression.

In conclusion, our findings suggest that dysregulated DNA methylation and active DNA demethylation in CLL B cells have a critical impact on disease progression and their association represents a major prognostic factor. This prognostic factor is independent from the other prognostic factors tested such as *IGHV* and CD38. Accordingly, stratifying CLL patients in epigenetic subgroups according to the 5-cyt derivative content, rather than to the analysis of the epigenetic regulators, can be helpful for improving CLL prognostic accuracy.

## MATERIALS AND METHODS

### Cells and sample preparation

Blood samples were collected from 55 untreated patients diagnosed with CLL according to the World Health Organisation (WHO) classification [46], and 17 healthy volunteers at the Brest University Medical School Hospital. In addition to the training cohort which includes the second time point samples from the longitudinal study, samples were collected for the first time point of the longitudinal study (*n* = 13) and for the validation cohort (*n* = 56). Consent was obtained from all individuals and the protocol approved by the Ethical Board at the Brest University Medical School Hospital (OFICE, November 26th, 2015; CRB Brest, collection 2008–214), in accordance with the Declaration of Helsinki.

Lymphocytes were isolated from peripheral blood mononuclear cells (PBMC) by Ficoll-Hypaque density gradient centrifugation (Eurobio, Courtaboeuf, France) and tumor B cells were further enriched using the Pan B-cell Isolation Kit (Miltenyi Biotec GmbH, Bergisch Gladbach, Germany). For control B cells, several purification protocols were conducted using either (1) the Pan B-cell Isolation Kit; the Naïve B Cell Isolation Kit II (non-target cells magnetically labeled with biotin-conjugated antibodies [Abs] against CD2, CD14, CD16, CD27, CD36, CD43, CD235a) and (3) the Switched Memory B Cell Isolation Kit (non-target cells magnetically labeled with biotin-conjugated Abs against CD2, CD14, CD16, CD36, CD43, CD235a, IgM, IgD) all from Miltenyi Biotec GmbH. For B1a cell subset isolation, purified CD19+ B cells from controls were further sorted on the basis of CD19 and CD5 surface expression with a MoFlow XDP cell sorter (Beckman Coulter, Brea, CA). Cell purity was assessed by fluorescence-activated cell sorting (FACS) analysis and was over 95% for B cells (CD19+), naïve B cells (CD19+, IgD+, CD27-) and switched memory B cells (CD19+, CD27+, IgD-). Purity of B1a cells (CD19+ CD5+) was > 98%. All FACS Abs used were purchased from Beckman Coulter.

### DNA sample preparation and global DNA 5-mCyt, 5-hmCyt, 5-CaCyt and 5-hmU levels assessment by ELISA

DNA was extracted from purified B cells using the Biosprint 15 DNA Blood Kit (Qiagen, Hilden, Germany). Next, DNA was quantified and its purity assessed using the NanoDrop 2000 Spectrophotometer (Thermo Fisher Scientific, Waltham, MA). An ELISA previously developed in the laboratory was used and adapted to measure global 5-mCyt, 5-hmCyt, 5-CaCyt and 5-hmU [48]. Briefly, high affinity microplates (Thermo 269620, Thermo Fisher Scientific) were pre-coated 90 min at room temperature (RT) with 100μl poly-L-lysine 0.01% (Sigma-Aldrich, St. Louis, MO) to attach DNA. Next, DNA samples adjusted at 2 ng/μl (for 5-mCyt and 5-hmCyt) and 1.5 ng/μl (for 5-CaCyt and 5-hmU) in carbonate/bicarbonate buffer 0.1M pH 9.6 were denatured at 95°C for 6 minutes, kept on ice 5–10 minutes and then 100 μl dispensed in each well, in duplicate. The plates were next incubated overnight at 4°C, three washes with phosphate buffer saline (PBS)-Tween 0.01% were performed and 200μl of PBS with bovine serum albumin (BSA) 1% were added in each well as a blocking solution. After 1 hour incubation at RT and extensive washing, 100 μl of mouse IgG anti-5-mCyt (diluted 1:5000 in PBS-BSA 1%), mouse IgG anti-5-hmCyt (1:1000), rabbit IgG anti-5-CaCyt (2μg/ml) or goat IgG anti-5-hmU (1:1000) were added and plates were incubated 2 hours at RT. All anti-cytosine derivative Abs were purchased from Abcam (Cambridge, UK). After 6 washes, 100 μl of alkaline phosphate-labelled goat anti-mouse, goat anti-rabbit or bovine anti-goat IgGs (Jackson Laboratory, Bar Harbor, ME), diluted at 1:5000 in PBS-BSA 1% were added and the plate was incubated for 1 hour at RT. After 3 washes, color was developed with 100μl p-nitrophenyl-phosphate (Sigma-Aldrich) diluted in carbonate/bicarbonate buffer 0.1M pH 9.6. Plates were kept at 37°C for 4 hours, and optical density (OD) determined at 405 nm using a Titertek Multiscan microplate reader (Flow laboratories, Rockville, MD). Each sample was tested in duplicate and non-specific background OD (duplicate wells without DNA) was subtracted from the corresponding test sample. For normalization, a reference sample (salmon sperm gDNA - Sigma Aldrich) was included on each plate and indexes calculated for 5-mCyt, 5-hmCyt, 5-CaCyt and 5-hmU using the ratio between the patient OD and the reference sample OD at 200ng/well (for 5-mCyt, 5-hmCyt) and 100 ng/well (for 5-CaCyt, 5-hmU).

### Mutational status of *IGHV*

According to the BIOMED-2 consortium guidelines [51], the *IGHV* gene mutation status was determined by sequencing after conducting a PCR multiplex amplification. Briefly, for multiplex PCR, 100 ng of genomic DNA, 0.25 µl of Ampli Taq Gold DNA Polymerase (Applied Biosystems, Foster City, CA), 10pmol of each primer, 0.2 mM dNTP Mix, 1.5 mM MgCl2, 1× PCR Buffer II, were adjusted to 50 µl with DNase/RNase free ultrapure distilled water. Next, PCR products were visualized on 2% agarose gel, and purified with ExoSAP-IT PCR product cleanup kit (Affymetrix, High Wycombe, UK). Finally, amplicons were sequenced with a Big Dye Terminator v3.1 cycle sequencing kit (Applied Biosystems). Results were analyzed with the database IMGT/HighV-Quest (The international ImMunoGeneTics information system, Montpellier) and a homology sequence > 98% defined an UM status [1].

### RNA sample preparation, reverse transcription and RTq-PCR.

RNA was extracted from purified B cells using the RNeasy Plus Micro Kit (Qiagen). Quantification and purity were assessed using the NanoDrop 2000 Spectrophotometer (Thermo Fisher Scientific). Next, RNA (300 ng) was reverse transcribed into cDNA using the Super Script III enzyme and random primers (Invitrogen Life Sciences, Carlsbad, CA). RTq-PCR was carried out in 20 µl mixtures containing 6 µl of template cDNA diluted 1/12 with DNase/RNase free ultrapure distilled water, 1X Power SYBR^®^ Green PCR Master Mix (Applied Biosystems) and 250nM of each primer (Table 4) using Applied Biosystems^®^ QuantStudio™ 7 Flex Real-Time PCR System. The PCR conditions were the same for all genes. All assays included a negative control, which consisted of the reaction mixture with no template. Comparison of cycle thresholds was completed with the 2^ΔΔCT^ method using *GAPDH* as an endogenous control.

**Table 4:** Primer sequences of genes used for real time quantitative PCR

Symbol	Gene description	Forward primer	Reverse primer
***GAPDH***	glyceraldehyde-3-phosphate dehydrogenase	TGCCCTCAACGACCACTTT	GGTCCAGGGGTCTTACTCCTT
***DNMT1***	DNA methyltransferase 1	CCTGTACCGAGTTGGTGATGGT	CCTTCCGTGGGCGTTTC
***DNMT3A***	DNA methyltransferase 3 alpha	CTCCTGTGGGAGCCTCAATGTTACC	CAGTTCTTGCAGTTTTGGCACATTCC
***DNMT3B***	DNA methyltransferase 3 beta	ACCACCTGCTGAATTACTCACGC	GATGGCATCAATCATCACTGGATT
***MBD2***	methyl-CpG binding domain protein 2	CCATGGAACTACCCAAAGGTCTT	CAGCAGATAAAAGGGTCTCATCATT
***MBD4***	methyl-CpG binding domain 4, DNA glycosylase	TCTAGTGAGCGCCTAGTCCCAG	TTCCAATTCCATAGCAACATCTTCT
***TET1***	tet methylcytosine dioxygenase 1	AATGGAAGCACTGTGGTTTG	ACATGGAGCTGCTCATCTTG
***TET2***	tet methylcytosine dioxygenase 2	AATGGCAGCACATTGGTATG	AGCTTCCACACTCCCAAACT
***TET3***	tet methylcytosine dioxygenase 3	TTGCGTCGAACAAATAGTGG	CCCGTGTAGATGACCTTCTC
***SAT1***	spermidine/spermine N1-acetyltransferase 1	GGTTGCAGAAGTGCCGAAAG	GTAACTTGCCAATCCACGGG

### Statistical analysis

The profile likelihood method using a Cox regression model of PFS and TFS was used in univariate analysis to determine the optimal threshold and stratify patients into two groups, and in multivariate analysis to compare risk factors. This analysis was computed using the Survival and SurvMisc R packages [9]. TFS and PFS analyses were performed using Kaplan–Meier curves and prognosis differences between groups were assessed with a log-rank test. Continuous data are described as mean ± standard error of the mean (SEM). Differences among groups were analyzed by one-way ANOVA in a non-parametric test, or the Fisher’s exact test for categorical data. Following normality and equality of variance tests, nominal values were compared to controls using the student’s *t* test or alternatively by using a nonparametric test (Mann-Whitney rank sum test). Paired samples were compared by Wilcoxon signed-rank tests and Pearson’s coefficient *r*^2^ for correlation analysis. *P* values under 0.05 were considered significant. Statistical analyses were performed using GraphPad Prism 7.0 (La Jolla, CA).

## SUPPLEMENTARY MATERIALS FIGURES


