# Supplementary material for: Alterations in DNA methylation/demethylation intermediates predict clinical outcome in chronic lymphocytic leukemia
Source: Oncotarget. 2017 Aug 9;8(39):65699–716. doi: 10.18632/oncotarget.20081 (PMC5630365; doi:10.18632/oncotarget.20081)
Supplement: Supplementary file 1 [file oncotarget-08-65699-s001.pdf]

Alterations in DNA methylation/demethylation intermediates predict clinical outcome in chronic lymphocytic leukemia

SUPPLEMENTARY MATERIALS

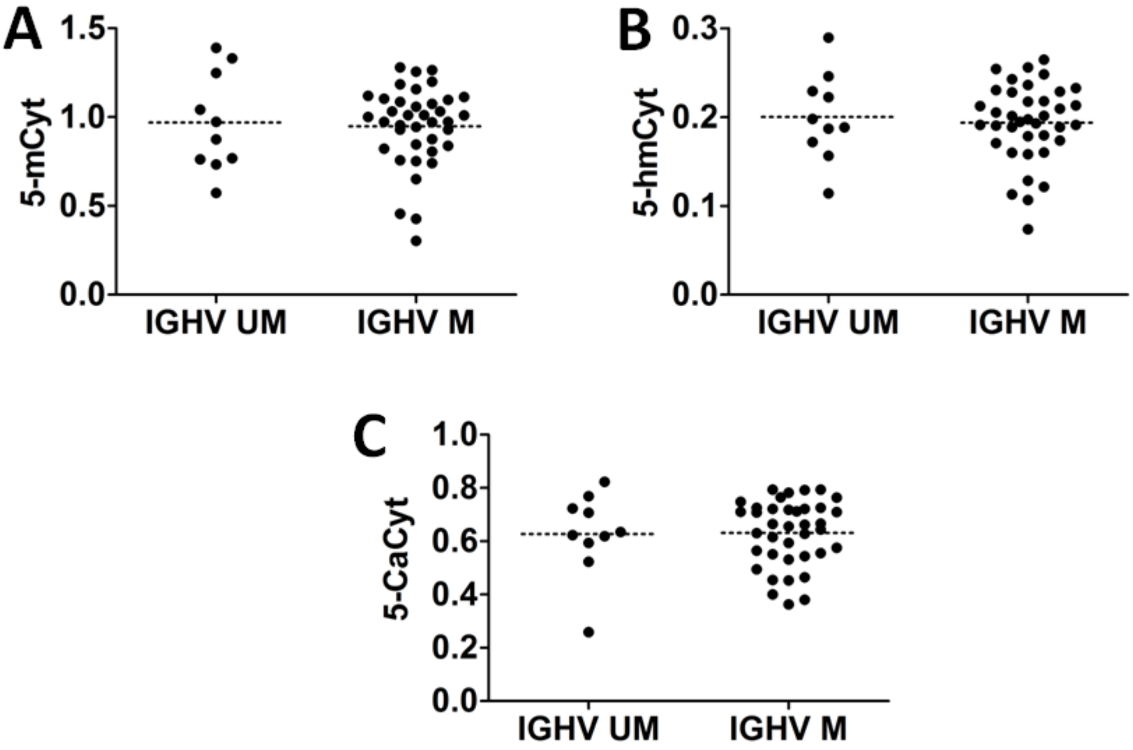

Supplementary Figure 1: Cytosine derivatives and *IGHV* mutational status. Chronic lymphocytic leukemia (CLL) patients tested for global DNA 5-mCyt (**A**), 5-hmCyt (**B**), 5-CaCyt (**C**) and immunoglobulin heavy chain variable region (*IGHV*) mutational status were divided into unmutated IGHV gene (UM > 98% homology from germline sequences) (n = 10) and mutated IGHV gene (M < 98% homology) (n = 38). The median value is indicated (dashed line) and statistical differences of cytosine derivatives levels between *IGHV* M and *IGHV* UM CLL patients, assessed using the non-parametric Mann-Whitney test, were not significant.

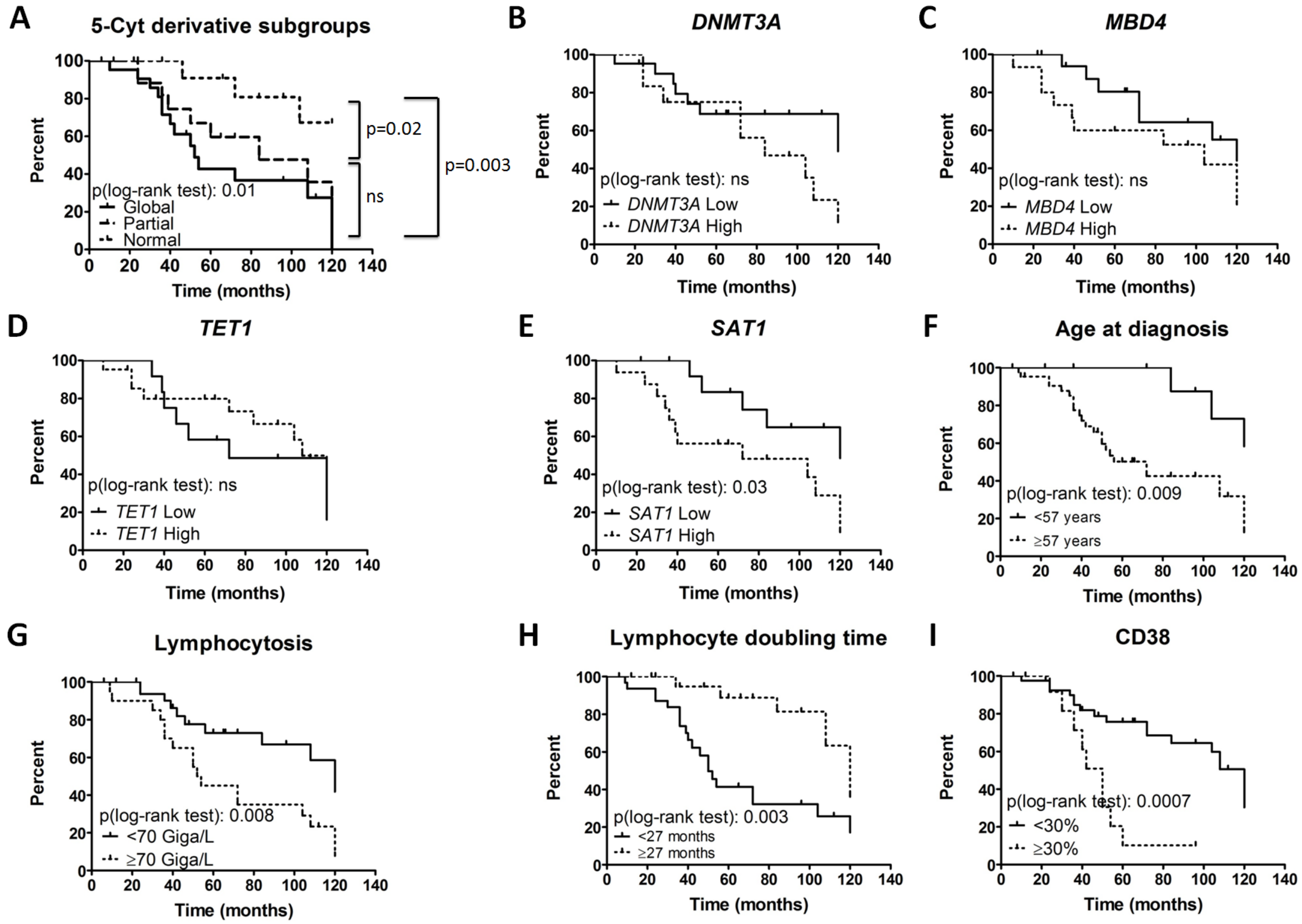

Supplementary Figure 2: Progression free survival (PFS) according to clinicobiological prognostic factors. Kaplan-Meier curves are displayed for 5-Cyt derivatives subgroups (**A**), *DNMT3A* (**B**), *MBD4* (**C**), *TET1* (**D**), *SAT1* (**E**), age at diagnosis (**F**), lymphocytosis (**G**), lymphocyte doubling time from diagnosis (**H**) and CD38 (**I**). Epigenetic regulator expression patterns were measured by real time quantitative PCR and normalized to *GAPDH*. The Cox regression model of PFS was used to identify the optimal cut-off level in order to dichotomize CLL patients into high *versus* low levels, except for CD38. Statistical differences between the curves were calculated using the log-rank test.
